# Brat Promotes Stem Cell Differentiation via Control of a Bistable Switch that Restricts BMP Signaling

**DOI:** 10.1016/j.devcel.2010.11.019

**Published:** 2011-01-18

**Authors:** Robin E. Harris, Michael Pargett, Catherine Sutcliffe, David Umulis, Hilary L. Ashe

**Affiliations:** 1 Faculty of Life Sciences, University of Manchester, Oxford Road, Manchester M13 9PT, UK; 2 Department of Agricultural and Biological Engineering and Weldon School of Biomedical Engineering, Bindley Bioscience Center, Purdue University, West Lafayette, IN 47907, USA

## Abstract

*Drosophila* ovarian germline stem cells (GSCs) are maintained by Dpp signaling and the Pumilio (Pum) and Nanos (Nos) translational repressors. Upon division, Dpp signaling is extinguished, and Nos is downregulated in one daughter cell, causing it to switch to a differentiating cystoblast (CB). However, downstream effectors of Pum-Nos remain unknown, and how CBs lose their responsiveness to Dpp is unclear. Here, we identify Brain Tumor (Brat) as a potent differentiation factor and target of Pum-Nos regulation. Brat is excluded from GSCs by Pum-Nos but functions with Pum in CBs to translationally repress distinct targets, including the *Mad* and *dMyc* mRNAs. Regulation of both targets simultaneously lowers cellular responsiveness to Dpp signaling, forcing the cell to become refractory to the self-renewal signal. Mathematical modeling elucidates bistability of cell fate in the Brat-mediated system, revealing how autoregulation of GSC number can arise from Brat coupling extracellular Dpp regulation to intracellular interpretation.

## Highlights

► Pumilio and Nanos translationally repress *brat* mRNA in germline stem cells ► Brat promotes differentiation by limiting Dpp signaling and cellular growth ► Brat acts with Pumilio to repress translation of the *Mad* and *dMyc* mRNAs ► Modeling shows Brat creates bistability and provides robust cell-fate control

## Introduction

**Figure 1:**
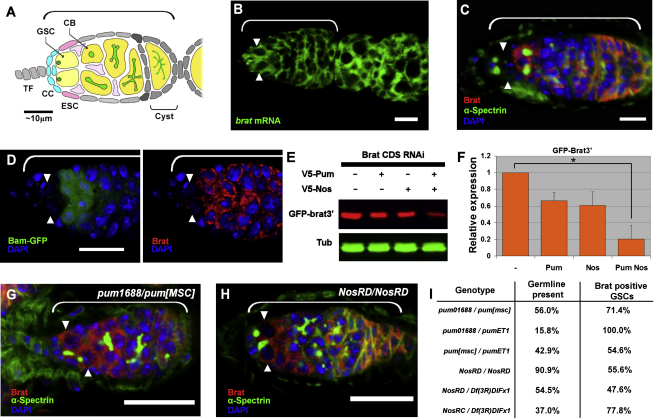
Brat Is Expressed in the Germline but Repressed by Pum-Nos in GSCs Germarium is indicated by the bracket in (A)–(D) and (G)–(H). Scale bars represent 10 μm (B–D) and 20 μm (G and H). (A) Structure of the *Drosophila* germarium and niche. TF, terminal filament; CC, cap cells; ESC, escort stem cell. (B) *brat* mRNA is expressed in the germarium, including GSCs (arrowheads). (C) Expression of Brat protein (red) is excluded from GSCs (arrowheads, spectrosome marked by α-Spectrin [green]). (D) Expression of Brat (red) is limited to CBs and differentiating cysts, marked by Bam-GFP expression (green). GSCs are labeled with arrowheads. (E) Representative western blot of *GFP-brat3′* repression by Pum-Nos in S2 cells. RNAi was used to knock down endogenous Brat. (F) Quantification of data in (E) displayed as the average of three biological repeats. Error bars are +SEM; repression by Pum/Nos compared to no regulator is statistically significant (^∗^p < 0.05). (G) Expression of Brat (red) in *pum* transheterozygous mutant ovaries, showing ectopic expression in GSCs (arrowheads). Ovaries were dissected immediately following eclosure to ensure the initial presence of germline cells. GSCs were identified by their position adjacent to niche cells and the fusome morphology and position. (H) As in (G), except that *nos* homozygous mutant ovaries were stained. (I) Table quantifying the presence of germline and number of GSCs ectopically expressing Brat protein in newly eclosed flies of different *pum* and *nos* mutant genotypes. Percentage of last column indicates proportion of Brat-positive GSCs out of total GSCs scored (n > 20). See also Figure S1.

Stem cells have the defining characteristic of being able to divide asymmetrically, producing a differentiating daughter cell while simultaneously renewing their own identity. The *Drosophila* ovarian germline serves as a paradigm for stem cell research due to its structural simplicity and accessibility (Kirilly and Xie, 2007). The *Drosophila* ovary consists of around 15–20 ovarioles—linear arrangements of developing eggs that originate from the anterior-most structure called the germarium. The germarium houses two to three germline stem cells (GSCs), from which the female germline derives. These GSCs are maintained by signals produced from various surrounding somatic cells, which together make up the ovarian stem cell niche (Figure 1A). The niche cells provide the key self-renewal signal Decapentaplegic (Dpp), which functions as a short-range signal to maintain the adjacent GSCs (Xie and Spradling, 1998) by directly repressing transcription of the key differentiation factor Bag of marbles (Bam) (Chen and McKearin, 2003a; Pyrowolakis et al., 2004; Song et al., 2004). When the GSC divides, one daughter cell remains within the niche, continuing to receive the Dpp self-renewal signal, whereas the other, the cystoblast (CB), moves posteriorly away from the source of the signal, leading to the derepression of Bam expression, which is both necessary and sufficient to cause germline differentiation (Ohlstein and McKearin, 1997). Loss of Dpp signaling allows cells to differentiate, whereas ectopic expression leads to tumorous expansion of GSCs (Chen and McKearin, 2003a; Xie and Spradling, 1998).

A number of intracellular factors that contribute to stem cell identity have also been characterized, including components of the miRNA pathway (Jin and Xie, 2007; Yang et al., 2007), as well as the translational repressors Pumilio (Pum) and Nanos (Nos) (Chen and McKearin, 2005; Forbes and Lehmann, 1998; Szakmary et al., 2005; Wang and Lin, 2004). It has been hypothesized that Pum and Nos maintain GSCs by repressing the translation of mRNAs encoding differentiation factors (Forbes and Lehmann, 1998; Szakmary et al., 2005; Wang and Lin, 2004), although the identity of these targets has yet to be elucidated. In CBs Nos is downregulated by Bam (Li et al., 2009), potentially allowing expression of these differentiation factors, thus contributing to the change of cell fate.

Cooperation of the Pum and Nos repressors has also been shown in different developmental contexts, including the embryonic germline where they regulate multiple aspects, such as pole cell migration and proliferation (Asaoka-Taguchi et al., 1999), and the postembryonic nervous system where they control morphogenesis of neuronal dendrites (Ye et al., 2004). Pum and Nos often function with an additional translational regulator Brain Tumor (Brat), as described for the repression of *hunchback* (*hb*) mRNA to pattern the early embryo (Sonoda and Wharton, 1999), and *paralytic* mRNA to control excitation of larval motoneurons (Muraro et al., 2008). Therefore, we investigated whether Brat also has a functional role in the ovary. Here, we provide evidence that Brat acts as a powerful differentiation factor within the germline via its limiting effects on the Dpp self-renewal pathway, allowing cells to robustly adopt distinct fates. Furthermore, our data demonstrate how stem cell progeny can rapidly commit to a differentiated fate despite proximity to a niche, a concept relevant to multiple stem cell systems.

## Results

### Brat Expression Is Limited to Differentiating Cells by Pum-Nos

We began by examining the expression of *brat* in the ovary. In situ hybridization using a *brat* anti-sense probe revealed that the *brat* transcript is expressed throughout the germline, including GSCs (Figure 1B), whereas immunohistochemistry showed that Brat protein is excluded from GSCs (Figure 1C). This is further confirmed by costaining with a Bam-GFP reporter that labels only differentiating cells (Chen and McKearin, 2003b) (Figure 1D). In the *bam* mutant, Brat protein is not present, but transcripts are still detected (see Figure S1 available online), suggesting that Brat may be expressed as part of, or in response to, a differentiation program, downstream of Bam. These observations also indicate that *brat* may be regulated in GSCs at the level of RNA translation. Pum and Nos are translational repressors known to be required autonomously in GSCs (Forbes and Lehmann, 1998). To investigate whether *brat* is a target of Pum-Nos repression, epitope-tagged constructs of *pum* and *nos* were coexpressed with a GFP reporter bearing the *brat* 3′UTR (GFP-brat3′) in *Drosophila* S2 cells. Because Brat protein is known to function with Pum-Nos in other contexts, and all three repressors are endogenously expressed in S2 cells (data not shown), RNAi targeting the *brat* coding region was employed to ensure that any regulation of the reporter was independent of Brat. The loss of endogenous Brat protein following RNAi treatment was confirmed by western blot (Figure S1). Analysis revealed that Pum-Nos cooperatively repress the GFP-brat3′ reporter expression (Figures 1E and 1F), but not GFP alone (Figure S1). Intermediate levels of repression by each regulator alone are attributed to the presence of endogenous Pum or Nos. To understand the relevance of this regulation in vivo, we examined various allelic combinations of *pum* mutant GSCs, which were found to ectopically express Brat protein (Figures 1G and 1I), indicating that *brat* is normally repressed by Pum in GSCs. Similarly, *nos* mutant GSCs also ectopically express Brat (Figures 1H and 1I). Moreover, when *nos* is ectopically expressed in anterior germline cells, *brat* repression is maintained in these cells, and the differentiation program is suppressed, producing ectopic GSCs (Figure S1). Taken together, these results indicate that the *brat* mRNA is regulated by Pum-Nos in the ovary, limiting its expression to CBs and developing cysts.

### Brat Restricts Cell Growth and Dpp Signaling in the Germline

**Figure 2:**
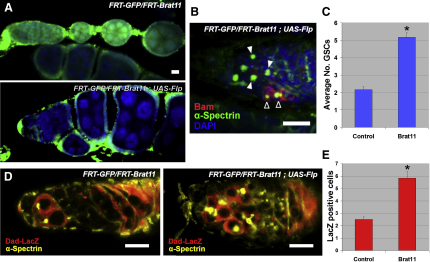
Mutation of *brat* Increases Growth and GSC Number (A) *brat^11^* mutant germline, indicated by a loss of GFP (lower panel), shows an increase in cell size compared to wild-type (upper panel). (B) A *brat^11^* germarium with increased GSCs, indicated by extra fusomes (green, solid arrowheads) and lack of Bam expression (red). Bam-positive CBs are indicated (open arrowheads). (C) Quantitation of GSC number in *brat^11^* mutant (n = 20); error bars are +SEM (^∗^p < 0.01). (D) Expression of *Dad-LacZ* (red) in wild-type and *brat^11^* mutant germaria, showing an expansion of *LacZ*-positive cells. (E) Quantitation displaying average number of Dad-lacZ-expressing germline cells (n = 6); error bars are +SEM (^∗^p < 0.0)1. See also Figure S2.

To investigate the function of Brat in the ovary, we generated a fly line in which the germline was homozygous mutant for the strong *brat^11^* allele, which truncates the protein and removes the vital NHL domain, shown to be necessary for protein-protein interaction (Arama et al., 2000). The *brat* mutant germline displayed an increased growth phenotype, with larger cells than wild-type (Figure 2A). The mutant germline also had more GSCs, as indicated by the presence of Bam-negative cells with round fusome organelles (Figures 2B and 2C). Analysis of two additional *brat* alleles reveals a similar expansion in GSC number (Figure S2). Because an increase in GSCs is seen when Dpp signaling is augmented in the germarium (Xie and Spradling, 1998), we investigated Dpp signaling in the *brat* mutant by analyzing expression of the Dpp target gene *Daughters against dpp* (*Dad*). In wild-type germaria a Dad-lacZ reporter is expressed strongly in GSCs, and at a lower level in CBs, reflecting Dpp-signaling activity (Casanueva and Ferguson, 2004) (Figure 2D). By contrast the expression domain of Dad-LacZ in the *brat* mutant is increased, confirming that the Dpp-signaling range is expanded (Figures 2D and 2E). These data indicate that Brat potentially has multiple functions within the differentiating germline, including cellular growth control and negative regulation of Dpp signaling.

### Brat Is a Potent Differentiation Factor in the Germline

**Figure 3:**
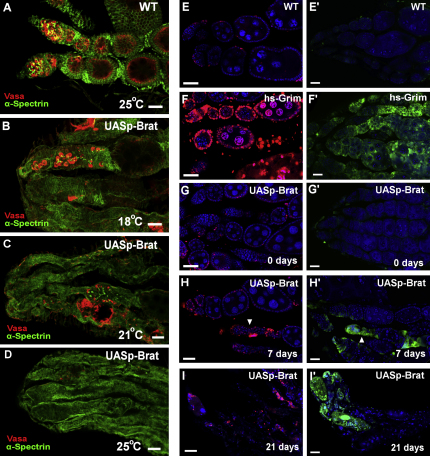
Ectopic Brat Strongly Promotes Differentiation Images in (A)–(D) are compiled Z stacks. Scale bars represent 10 μm. (A) Wild-type germarium stained for Vasa (red) and α-Spectrin (green) to mark cytoplasm and fusome structures of germline cells, respectively. (B) Ectopic expression of Brat in the germline reduces number of germline cells, in addition to disruption of overall morphology of the ovary. Several germline-less ovarioles are present. (C) Ectopic Brat expression at higher levels through growth at 21°C enhances the loss of germline. (D) Ectopic expression at 25°C results in ovaries lacking germline cells. Analysis of cell death in Brat transgene expression using TUNEL assay to indicate apoptosis (red) (E)–(I) and viability staining to reveal other cell death, including necrosis (green) (E′)–(I′). Wild-type ovaries show little background staining for either cell death marker (E and E′), whereas ovaries from flies expressing the apoptosis-promoting gene *grim* under control of the heat shock promoter demonstrate widespread cell death following its induction (F and F′). Ovaries expressing the UAS-*brat* transgene initially show no cell death above background (G and G′), whereas after 7 days only apoptosing somatic cells are detected at the tip of ovarioles with maturing egg chambers, suggesting that they are remnants of germaria that have now emptied (H and H′, arrowheads). Following 21 days, most germaria are empty, and no egg chambers remain, leaving only apoptosing somatic cells (I and I′). No increase in germline cell death was detected at any stage. See also Figure S3.

To further clarify the function of Brat, we ectopically expressed it specifically in the germline using the GAL4-UAS system and staining for the germline marker Vasa (Figure 3A). When Brat is ectopically expressed in this way, the number of germline cells present is severely diminished, whereas other defects such as missing egg chambers and mispackaged cysts are evident (Figures 3B and 3C; Figure S3). When the activity of GAL4 is increased by raising the growth temperature, the phenotypes become increasingly severe, ultimately resulting in a complete loss of germline and female sterility (Figure 3D; Figure S3). A TUNEL assay reveals no increase in programmed cell death of germline cells over time (Figures 3E–3I), whereas viability staining confirmed an absence of cell death by other mechanisms, such as necrosis (Figures 3E′–3I′). Both assays detected some dying somatic cells at the anterior tip of empty germaria (Figures 3H–3I′), suggesting that an intact germline may be required to maintain the germarium structure. Together, these results reveal Brat to be a powerful differentiation factor within the germline.

### Brat Requires Interaction with Pum to Promote Differentiation

**Figure 4:**
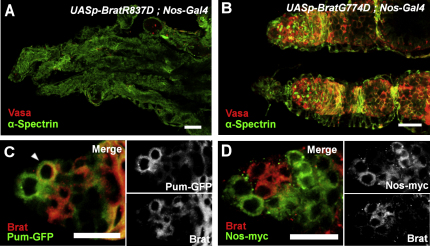
Brat Functions Together with Pum in the Germline Images (A) and (B) are compiled Z stacks. Scale bars represent 10 μm. (A) Germaria of flies ectopically expressing Brat^R837D^ that cannot interact with d4EHP phenocopy wild-type ectopic expression, whereas those expressing Brat^G774D^ (B), which cannot interact with Pum, have a reduced phenotype, indicated by the presence of GSCs and a developing germline (Vasa, red). (C) Codetection of Brat and Pum shows that they overlap in CB cells (arrowhead). Separate channels show individual staining for clarity. (D) Codetection of Brat and Nos shows that they are expressed in reciprocal domains, suggesting that Pum-Brat activity is independent of Nos in differentiating cells. Separate channels are shown. See also Figure S3.

Brat interacts with the d4EHP and Pum translational repressors to repress the *hb* mRNA in the embryo (Cho et al., 2006; Sonoda and Wharton, 2001). To unravel the molecular mechanism of Brat-mediated differentiation in the germarium, we ectopically expressed mutant forms of the Brat protein that either cannot interact with d4EHP or Pum. These two *brat* mutant transgenes are expressed at approximately equivalent levels to the wild-type transgene (Figure S3). Expression of Brat^R837D^, which cannot interact with d4EHP (Cho et al., 2006), resulted in the same loss of germline seen for ectopic expression of wild-type Brat (Figure 4A), suggesting that d4EHP interaction is dispensable for Brat's function in the ovary. The ovaries of initially eclosed flies expressing wild-type Brat or Brat^R837D^ had few developing egg chambers and no mature embryos, whereas after a period of 21 days, only empty germaria remained (Figure S3). Conversely, ovaries expressing the Brat^G774D^ mutant, which has a reduced ability to bind Pum (Sonoda and Wharton, 2001), have a less-severe phenotype (Figure 4B), indicating that Brat requires an interaction with Pum in order to promote differentiation. These flies eclosed with ovaries bearing intact germlines, which persisted over 21 days and produced mature embryos, although these were rarely laid for unknown reasons (Figure S3). Because Pum is expressed in GSCs and also coincides with Brat expression in CBs (Figure 4C), this led us to consider the possibility that Brat is repressed by Pum-Nos in GSCs but acts in conjunction with Pum in CBs. Evidence shows that Nos is downregulated in CBs by Bam (Li et al., 2009), implying that Nos is not involved in this Pum-Brat interaction. When Brat and Nos are visualized simultaneously, they were found to be expressed in reciprocal patterns (Figure 4D), supporting the idea that Brat interacts with Pum independently of Nos in CBs, thus establishing a distinct repression complex. Because Brat protein expression overlaps that of Bam (Figure 1D), and is downstream of it (Figure S1), it is possible that Bam downregulates Nos to allow Brat expression, which then forms a new complex with Pum that acts in favor of differentiation.

### Pum-Brat Represses the Dpp Signal Transducer Mad and dMyc

**Figure 5:**
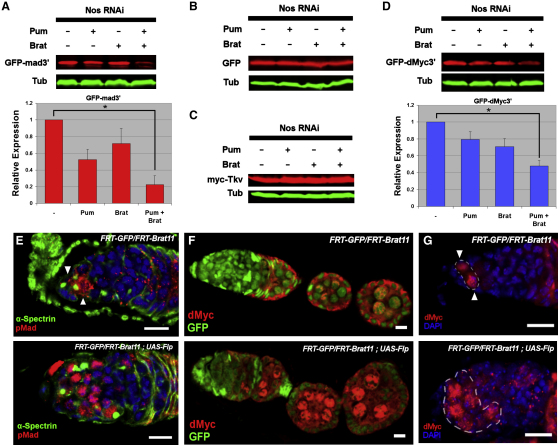
Brat Represses Mad and dMyc in the Ovary (A) Representative western blot showing Pum-Brat regulation of *GFP-mad3′* in S2 cells. RNAi ensures that effects are independent of Nos. The intermediate level of repression by Pum is attributed to the contribution of endogenous Brat protein, and vice versa. Graph shows quantification of independent biological repeats (n = 4). Error bars are +SEM; repression by Pum-Brat compared to no regulator is statistically significant (^∗^p < 0.05). (B and C) Western blot showing that GFP without 3′UTR (B) and epitope-tagged Tkv (C) are not regulated by Pum or Brat. (D) Pum-Brat regulation of *GFP-dMyc3′* reporter, graph as in (A), repression by Pum-Brat compared to no regulator is statistically significant (^∗^p < 0.05). (E) Expression of pMad (red) in wild-type (top) and *brat* mutant (bottom) germaria, showing expansion of activated Mad in germline cells mutant for Brat. (F) dMyc expression (red) in a *brat* mutant ovariole (bottom) begins earlier and is at a higher level compared to a wild-type ovariole (top). (G) Detailed view of dMyc expression (red, outlined) at the anterior of wild-type germaria, showing high level of expression in GSCs (top), and an expansion of this expression in the *brat* mutant (bottom). Scale bars represent 10 μm (E–G). See also Figure S4.

Having shown that Brat acts to promote differentiation, we next examined the potential targets of Brat repression. Because loss of *brat* function leads to expansion of Dpp signaling (Figure 2D), we investigated whether Dpp pathway members could be subject to repression by Pum-Brat. Epitope-tagged Pum and Brat constructs were transfected into *Drosophila* S2 cells, along with reporters or tagged ORFs of the Dpp-signaling transducer *Mothers against decapentaplegic* (*Mad*) and the Dpp receptor *thickveins* (*tkv*). Because S2 cells express *pum*, *nos*, and *brat* at detectable levels (data not shown), *nos* RNAi was used to ensure that the results reflect *nos*-independent regulation and, therefore, approximate the in vivo situation. The RNAi effectively suppresses Nos expression (Figure S4). Western blot analysis reveals that a GFP construct bearing the *Mad* 3′UTR (GFP-Mad3′) was significantly repressed when cotransfected together with both Pum and Brat (Figure 5A), whereas GFP alone or the *Mad* coding sequence was not (Figure 5B; Figure S4). Removal of *nos* RNAi reveals the same pattern of repression (Figure S4), although the weaker effect may indicate that Nos and Brat compete for Pum. A tagged *tkv* construct bearing both UTRs showed no repression (Figure 5C), suggesting that the Dpp receptor is not a Pum-Brat target. Because mutation of *brat* also caused a significant growth phenotype, we tested the growth regulator *dMyc*, which has been implicated as a Brat target in neural cells (Betschinger et al., 2006). A reporter bearing the *dMyc* 3′UTR (GFP-dMyc3′) showed significant repression (Figure 5D), indicating that Pum-Brat can also regulate dMyc-mediated cellular growth. Because expression of all reporters is under the control of the *actin* promoter, it is unlikely that the repression is at the transcriptional level but, instead, points to posttranscriptional regulation, consistent with it being conferred by the presence of the UTRs.

To confirm that these mRNAs are authentic targets of Brat repression in vivo, Mad and dMyc proteins were visualized in the *brat* mutant germline. In the absence of a suitable Mad antibody, the expression of activated Mad (pMad) was examined. Wild-type pMad is limited to GSCs and CBs at lower levels, reflecting Dpp-signaling activity as described previously (Song et al., 2004) (Figure 5E). In *brat* mutant germaria, pMad protein distribution is expanded (Figure 5E), consistent with Mad being a target of Brat repression. Examination of dMyc protein in wild-type germaria reveals moderate levels of nuclear expression beginning after 16-cell cyst encapsulation (Figure 5F), and high levels in GSCs with reduced expression in CBs (Figure 5G), as described previously (Rhiner et al., 2009). In the *brat* mutant germline, expression of dMyc occurs earlier and is more evident in the nucleus, whereas at the anterior germarium, expression is present in additional cells (Figures 5F and 5G). Therefore, our data suggest that Brat repression limits interpretation of the Dpp signal, in addition to restricting dMyc-mediated cellular growth.

### Brat Provides Bistability and Contributes to Cell Competition

**Figure 6:**
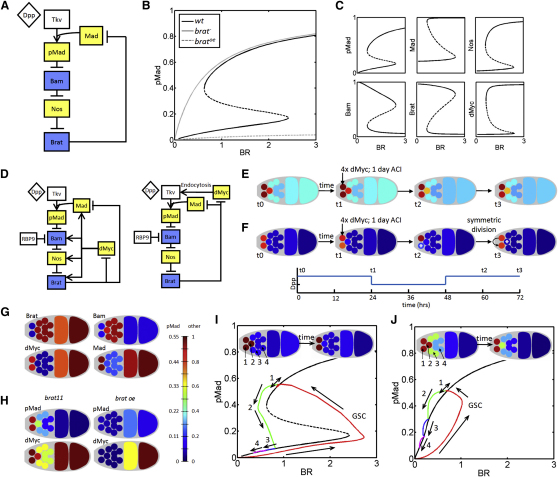
Brat-Mediated Repression of Mad and dMyc Leads to Bistability (A) Network diagram implemented in modeling studies, with components supporting self-renewal (yellow) or differentiation (blue). (B) Equilibrium distribution for pMad in wild-type, showing the two stable states (solid-black portions) and one unstable (dashed portion). Levels of pMad in Brat ectopic expression (lowest dashed line) and *brat* mutant (solid gray) illustrate the lack of bistability under these conditions. (C) Levels of pMad and other intracellular signal transducers and modifiers in the single-cell local model. (D) Comparison of network diagrams representing the “synthesis” model network (left) and the alternative “endocytosis” dMyc model (right). (E) Time course of the “synthesis” model. Colors indicate pMad levels (see color scale in next panel). From a wild-type germarium (t0), a clone expressing *4xdMyc* is induced and allowed to stabilize (t1). Clone exhibits slightly higher levels of pMad, without affecting levels in adjacent cells. After a transient decrease in Dpp ligand, both the clone and wild-type GSC recover the self-renewal state, remaining as GSCs (t2). (F) Time course of the “endocytosis” model. The removal of local Dpp by the *4xdMyc* cell lowers pMad signaling in an adjacent GSC (t1), causing this cell to be more susceptible to small perturbations in the level of Dpp, eventually leading to its differentiation (t2, asterisked cell). The remaining *4xdMyc* GSC takes over the germarium (t3, arrow), demonstrating symmetrical division. (G) Model protein distributions for Brat, Bam, dMyc, and Mad in a wild-type germarium (see color scale). (H) pMad, dMyc, and Bam in the *brat* mutant (*brat^11^*) and ectopic *brat* expression (*brat oe*) germaria, which mirror in vivo levels. For pMad wild-type, see (F, t0). (I) pMad expression during differentiation of GSCs predicted by the 3D spatiotemporal model. Immediately after division of a GSC (position 1), the pre-CB cell levels of pMad signaling drop (position 2), and signaling declines steadily and remains low in the two to four cell cysts (position 3). The newly formed CB follows trajectory 2, whereas the more posterior cyst follows trajectory 3 in the lower pMad state. Dedifferentiation of a cell, during repopulation of an emptied niche for example, is represented by the red line. (J) pMad expression during GSC differentiation in the 3D model of a *brat* mutant, representative of a system lacking bistability. pMad signaling is high in GSCs (position 1) and declines smoothly in the CB and two to four cell cysts (positions 2–4). See also Figure S5.

To assess the potential of the Brat-mediated network to produce a necessary sharp transition between adjacent self-renewing and differentiating cells, we generated a local, single-cell mathematical model of the intracellular network based on our experimental data (Figure 6A). This simplified, local model includes Brat-mediated translational repression of *Mad* and assumes sufficient levels of both intracellular Pum and Med. The model confirms that Brat-mediated repression of *Mad* can confer bistability to pMad and other components of the network (Figures 6B and 6C) and that this bistable behavior is largely insensitive to the precise parameter values (Figure S5). Therefore, the intracellular network allows two adjacent cells such as the GSC and CB to have equivalent levels of bound and signaling Dpp ligand, yet distinct levels of pMad and other intracellular components. The GSC or CB fate adopted by each cell depends on the cellular machinery, the levels of Pum, Brat and Mad, as well as the history of active signaling in the cell. Loss or ectopic expression of Brat in the model abolishes this bistability, and cells become either ultrasensitive or refractory to extracellular Dpp (Figure 6B), which is consistent with the observations in Figures 2 and 3.

To investigate Brat-mediated differentiation in the niche space and cell-to-cell competition, we developed a 3D spatiotemporal model of an idealized germarium. The 3D model contains two volumetric compartments: extracellular and intracellular, and includes chemical reactions and exchange of molecules between each individual cell's surface and the intracellular compartment. This allows us to investigate cell responses to changing conditions in the germarium, as well as interactions between cells, such as competition for Dpp in the niche space. The 3D model also includes additional elements, such as RBP9, which regulates Bam in developing cysts (Kim-Ha et al., 1999), and incorporates the repression of *dMyc* by Brat (Figures 5D and 5G; Figure S5). Because the mechanism of dMyc action in the germarium is unknown, we used the model to investigate two putative functions for dMyc (Figure 6D). The first “synthesis” model involves a network whereby dMyc upregulates the overall intracellular protein synthesis rate, consistent with the previously observed role of dMyc in cellular growth control (Grewal et al., 2005). The second “endocytosis” model is based on data suggesting that cells with higher dMyc levels outcompete cells with less dMyc, potentially by increasing uptake of the Dpp ligand (Moreno and Basler, 2004; Rhiner et al., 2009). In both models the networks lead to bistability and the capacity for sharp delineation between adjacent cells. Each network was tested by modeling a situation in which one of the two GSCs expressed ectopic *dMyc* (4x*dMyc*), which has been shown to lead to GSC competition and niche takeover by the 4x*dMyc* cell in vivo (Rhiner et al., 2009). In the “synthesis” model the introduced *4xdMyc* cell exhibits no significant change in pMad level, or only a small increase for some parameter sets, and no changes are evident in the adjacent cells (Figure 6E, t1). When the Dpp level is transiently reduced, the 4x*dMyc* clone does differ from the adjacent GSC (Figure 6E, t2). However, modification of the intracellular network alone cannot confer a competitive advantage to the cell, and on return to normal Dpp levels, both cells return to the previously observed steady state (Figure 6E, t3). In this network the 4x*dMyc* clone cannot outcompete wild-type GSCs, inconsistent with the in vivo observations (Rhiner et al., 2009). In contrast the “endocytosis” model reveals that although pMad levels also do not substantially increase in the 4x*dMyc* cell, signaling in the adjacent GSC diminishes (Figure 6F, t1). The low dMyc expressing GSC is susceptible to small perturbations in the level of Dpp such that it will differentiate following a transient loss of Dpp (Figure 6F, t2), allowing the 4x*dMyc* clone to take over the niche and germarium via symmetric division (Figure 6F, t3). Thus, this network exhibits effective competition and predicts that it may occur by limiting the amount of Dpp ligand available to the “loser” cell, rather than through an increase of signaling in the “winner” 4x*dMyc* cell. The comparison of the two networks demonstrates how simple upregulation of endocytosis can in principle lead to competition of GSCs for Dpp or other niche signals, although additional experiments aimed at measuring Dpp uptake in GSCs and CBs will be required to directly test this model.

Because the “endocytosis” model more faithfully reflects GSC behavior in vivo, its validity was further considered by examining the spatial expression of network components. The expression patterns generated are consistent with our experimental observations in both wild-type germaria (Figure 6G) and under conditions of altered Brat levels (Figure 6H). The model was then used to explore the signaling dynamics during division and differentiation of a GSC. Our analysis reveals that immediately following GSC division, the daughter cell, which we assume contains initially high pMad and low Brat levels inherited from the mother cell, rapidly evolves to the low Dpp-signaling state (Figure 6I, positions 1–2). The rapid evolution of pMad in the CB can be attributed to two dominant and directly related processes: lower Dpp levels at the distal position of the CB relative to the GSCs, and competition between the CB and GSCs for available Dpp. In general, we find that the cell in position 2 is sensitive to Dpp production rates and the effective diffusivity of Dpp, which may explain the experimental observation of two to three GSCs per niche in wild-type germaria.

We also tested whether the model could recapitulate other behavior of the stem cell system seen in vivo, including the reoccupation of an empty niche by the dedifferentiation of developing cysts. If no GSCs are present, resulting in an absence of competition, the increase in available Dpp is sufficient to reverse the differentiated fate of a CB (Figure 6I, red line to position 1), restoring the GSC population. To test this phenomenon for cells far from the Dpp source, we developed a 2D cell array model and found that in a niche devoid of cells and a germarium with a cyst-fated cell in the posterior region, the distribution of Dpp automatically adjusts and switches the differentiating cell into a GSC-like cell (unpublished data). Overall, these data, together with the predicted function of dMyc in endocytosis, are consistent not only with the cell competition data obtained (Rhiner et al., 2009) but also the suggested mechanism of GSC replacement through symmetric division of a single GSC (Xie and Spradling, 2000) and the repopulation of an empty niche (Kai and Spradling, 2004), providing insights and testable hypotheses for a better understanding of the underlying interactions responsible for these behaviors in vivo.

Finally, the case of a *brat*-null mutant was considered to observe signaling dynamics in a system that is sensitive to Dpp signaling but does not exhibit the bistability provided by Brat (Figure 6J). In the absence of Brat, sensitivity to Dpp signaling is established throughout the system, consistent with an increase in GSC-like cells in the germarium, as seen in vivo (Figure 2B). Cells that are far from the Dpp source will eventually begin to differentiate but are much more susceptible to dedifferentiation (Figure 6J). Thus, the Brat-mediated repression of *Mad* and *dMyc* provides a robust autoregulatory module that is essential for maintaining stable and discrete cell fates during the transition from self-renewing to differentiated identity. Without Brat, this sharp delineation is lost, leaving the differentiation process vulnerable to perturbations in the Dpp signal.

## Discussion

**Figure 7:**
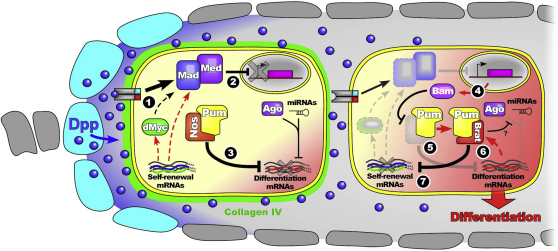
Brat's Role in the Ovarian Stem Cell System Schematic showing the function of Brat in the germline. See text for details.

Overall, our data show that the decision of a stem cell to self-renew or differentiate depends on feedback generated by a network of translational repressors (Figure 7). When anchored to the niche, the GSC receives high levels of Dpp signal due to its proximity to ligand-producing cells, in addition to the effects of a competitive mechanism, modeled here as increased Dpp-ligand uptake, resulting from high dMyc expression (Figure 7, 1). These factors, in conjunction with other molecular players that augment Dpp signaling (see below), silence transcription of Bam (Figure 7, 2), allowing Pum-Nos to repress mRNAs encoding differentiation factors, including the *brat* mRNA (Figure 7, 3). Following GSC division, a transient loss of Dpp signaling is sufficient to initiate a chain of events that ultimately locks the CB into a differentiation fate. A reduction in Dpp signaling allows derepression of Bam expression in the CB (Figure 7, 4). Bam then downregulates Nos (Figure 7, 5) to subsequently allow the translation of differentiation-promoting factors, including Brat (Figure 7, 6). Brat interacts with Pum to form a distinct translational repression complex with altered specificity, which targets self-renewal mRNAs, including those of *Mad* and *dMyc* (Figure 7, 7). Downregulation of signal transduction by Brat's repression of *Mad* can explain at the molecular level the previously reported genetic evidence showing that Bam limits Dpp signaling downstream of its receptor in the ovary (Casanueva and Ferguson, 2004) because Brat is expressed downstream of Bam. The loss of *Mad* in GSCs has been shown to cause their differentiation (Xie and Spradling, 1998), whereas the loss of just one copy of *Mad* can reverse the effect of Dpp ectopic expression (Xi et al., 2005), illustrating the acute sensitivity of germline cells to changes in Dpp pathway components and, hence, Brat's impact on cellular fate. Brat repression of Mad and dMyc diminishes the CB's ability to respond to the Dpp signal, committing the cell to the differentiation program.

The regulation of Dpp transduction is one level at which Dpp signaling is regulated within the ovarian stem cell system. The production and availability of the ligand depend upon transcriptional control in niche cells mediated by JAK-STAT signaling (Lopez-Onieva et al., 2008; Wang et al., 2008a), whereas spatial regulation is provided by extracellular elements such as type IV collagens that sequester Dpp ligand (Wang et al., 2008b), and the glypican Dally that potentiates local Dpp signaling (Guo and Wang, 2009; Liu et al., 2010), thus contributing to Dpp's limited availability. The action of Brat ensures that ligand present in the vicinity of CBs is transduced at a limited rate. This is complemented by dSmurf-mediated degradation of Mad (Casanueva and Ferguson, 2004; Chen and McKearin, 2005), and the control of expression and localization of the Dpp receptor Saxophone by miR-184 in differentiating cells (Iovino et al., 2009). Thus, multiple mechanisms are employed to restrict Dpp-signaling activity in the germarium, consistent with the essential requirement to downregulate Dpp signaling to ensure proper oogenesis. However, whereas the germarium contributes to the spatial control of Dpp, Brat-regulated mechanisms, including the repression of *dMyc*, provide a link between extracellular regulation and intracellular interpretation.

The interactions we have identified and modeled form a bistable network, which demonstrates multiple behaviors that are observed in vivo. The bistability generates a large difference between the minimum amount of Dpp required to maintain self-renewal and that needed to transition from differentiation to self-renewal. This effect affords a robust mechanism, in concert with cell competition, to ensure a constant population of GSCs because the balance between Dpp secretion and utilization is sufficient to support only two to three GSCs. Our modeling data suggest that dMyc-mediated competition can be explained by a role for dMyc in promoting endocytosis of Dpp. However, because the link between increased dMyc and elevated pMad levels is molecularly undefined, we cannot exclude other possibilities, such as influences of dMyc on pMad stability or maintenance of its phosphorylated state. In addition to the effect of dMyc, physical niche interactions, such as niche adherence and forces from crowding, have also been suggested as a competitive interaction downstream of Bam expression (Jin et al., 2008). Interestingly, niche adherence in GSCs, mediated by E-cadherin, has been observed to be independent of dMyc (Jin et al., 2008; Rhiner et al., 2009), indicating that these two potential competition mechanisms are not coupled at the level of dMyc expression. It is likely that there are multiple parallel competitive mechanisms downstream of Bam, which could potentially be mediated by Brat, and extension of the model will contribute to their delineation in the future.

Our data are consistent with Pum being a member of distinct translation repression complexes that target different pools of mRNAs. Evidence suggests that the recruitment of Pum corepressors is both context and target dependent. The well-documented Pum-Nos-Brat complex that targets the *hb* mRNA in the embryo (Sonoda and Wharton, 2001) does not appear to play a role in the germarium because Nos and Brat have mutually exclusive expression patterns. Although there are precedents for a Pum-Nos complex functioning in the absence of Brat, e.g., in the repression of the *cyclinB* (*cycB*) mRNA in embryonic pole cells (Kadyrova et al., 2007; Sonoda and Wharton, 2001), to our knowledge, a Pum-Brat complex has not been described previously. Because Nos functions with Pum to co-recruit Brat to the Pum-Nos-Brat complex, it is likely that in the absence of Nos, another factor takes on this role in CBs. One possibility is that Bam and its binding partner BGCN are responsible for this function because they have been implicated in translation repression in CBs (Li et al., 2009) and can interact with Pum (Kim et al., 2010). Dissection of elements within the mRNA 3′UTRs conferring specificity of translation repression by either the Pum-Nos or Pum-Brat complexes will ultimately be informative in terms of understanding mRNA selection, although potentially challenging due, in part, to the loose binding consensus of Pum (Gerber et al., 2006) and its binding promiscuity (Gupta et al., 2008).

Brat belongs to the NHL domain family that also includes the *Drosophila* protein Mei-P26, which, like Brat, acts to promote ovarian germline differentiation (Neumuller et al., 2008). Both Brat and Mei-P26 have higher expression in CBs than GSCs, yet this is achieved by distinct mechanisms. For Brat, Pum-Nos repression of the mRNA ensures that its expression is limited to CBs and not GSCs, whereas the expression of Mei-P26 is stimulated in CBs by the binding of Vasa to a U-rich region of the *mei-P26* mRNA, which then potentially recruits the essential translation activator eIF5B (Liu et al., 2009). In addition the miRNA pathway has been implicated in repression of Mei-P26 in the *Drosophila* wing (Herranz et al., 2010), and because the miRNA pathway is active in GSCs (Park et al., 2007), it may serve to negatively regulate Mei-P26 levels in these cells. In addition to the distinct mechanisms used to actively limit Brat or Mei-P26 protein to the CB, it appears that each paralog also mediates differentiation through contrasting mechanisms. Although our work shows that Brat acts via direct repression of self-renewal promoting mRNAs, Mei-P26 is thought to function through inhibition of the miRNA pathway, affecting regulation of differentiating-promoting target mRNAs in CBs and differentiating cells (Neumuller et al., 2008). Mei-P26 interacts with the miRNA pathway effector Argonaute (Ago1), although how this interaction affects the miRNA pathway is currently unclear. Mei-P26 shares significant domain homology with the *C*. *elegans* protein Ncl-2 (Glasscock et al., 2005), a putative E3-ubiquitin ligase, suggesting that Mei-P26 could act via ubiquitination to downregulate either Ago1 directly or another component of the miRNA pathway. Indeed, mammalian homologs similarly control cell fate through ubiquitination and miRNA regulation, such as the mouse ortholog mLin41 that antagonizes Ago2 by ubiquitination (Rybak et al., 2009), and TRIM32 that regulates miRNA level in neuroblast progenitors and additionally ubiquitinates c-Myc (Schwamborn et al., 2009). Although Brat can also bind Ago (Neumuller et al., 2008), it is not known whether Brat functions through miRNA regulation in the germarium (Figure 7). However, Brat lacks the RING finger domain known to provide ubiquitin-ligase activity (Glasscock et al., 2005), potentially accounting for the contrasting mechanisms of regulation described for Brat and Mei-P26 in the control of germline differentiation.

Brat has been previously identified in an additional stem cell system of *Drosophila* as a key regulator of neuroblast progeny differentiation during development of the larval brain (Bello et al., 2006; Betschinger et al., 2006). Together with our findings, these data could suggest a global function for Brat in promoting cell fate changes in stem cell systems. Brat orthologs have been identified in other species and have also been implicated in disease, including neurodegeneration in mice (Balastik et al., 2008) and formation of brain tumors in humans (Boulay et al., 2009). Thus, our study will serve as a platform for understanding differentiation in mammalian stem cell systems as well as related genetic diseases.

## Experimental Procedures

### Fly Stocks

Fly stocks used were: *yw^67c23^* as the initial stock to generate transgenics; *Dad-LacZ* (Tsuneizumi et al., 1997); *GFP-Pum* (Buszczak et al., 2007); *Nos-myc* (Verrotti and Wharton, 2000); *Gal4-Nos:VP16, pum^Msc^, pum^01688^, pum^ET1^, UAS-Flp* (Bloomington Stock Center); *Bam-GFP* (Chen and McKearin, 2003b); *nos^RC^*, *nos^RD^* (a gift from R. Baines); Df(3R)Dl^FX1^ (Forbes and Lehmann, 1998); and *brat^11^* (Frank et al., 2002). Transgenic flies were generated by standard *P* element-mediated transformation using the pUASp vector. *brat* mutants were generated by site-directed mutagenesis using QuikChange (Stratagene).

### Generation of *brat* Clonal Germlines and Immunofluorescence

Clones were generated by FLP/FRT recombination. Flies of genotype *Gal4-Nos:VP16*/*+* ; *FRT-brat*/*FRT-GFP* ; *UAS-FLP*/+ were dissected; clones were confirmed by loss of GFP. As a control *UAS-FLP* was omitted. For immunofluorescence, adults were dissected after maturing on yeast/apple juice agar plates, and ovaries were fixed and stained using standard techniques (detailed methods available on request). Antibodies used are detailed in Supplemental Experimental Procedures.

### Tissue Culture and Western Blot

S2 cells were cultured in Schneider's Modified *Drosophila* Media (Invitrogen), supplemented with 10% FBS and 1% Pen/Strep. DNA constructs were transfected using Effectene (QIAGEN) according to manufacturers' instructions, and cells harvested following 3 days expression. Western blot was performed using standard methods and detected using Li-Cor Infrared detection system. Primary antibodies were used at 1:2000 dilution: mouse anti-HA (Santa Cruz); mouse anti-Myc (Santa Cruz); rabbit anti-Flag (Roche); mouse anti-V5 (Abcam); rabbit anti-GFP (Abcam); rabbit anti-Brat (Sonoda and Wharton, 2001); and mouse anti-Tubulin (Roche). Secondary antibodies were used 1:10,000 dilution: IRDye 800CW goat anti-mouse and IRDye 680LT goat anti-rabbit (Li-Cor).

### Statistical Analysis

Western blot IR signals were quantified using Li-Cor Odyssey software. Error bars were generated from the SEM of at least three separate biological repeats with Student's t test used to evaluate significance.

### Mathematical Modeling

For the local cell model, we developed a set of coupled, nonlinear ordinary differential equations, which are given along with parameters in the Supplemental Experimental Procedures. The core model is a simplified form of the known interaction network in BMP signaling. This model considers only BMP-bound receptors, Mad, Bam, Nos, and Brat, with a double-negative feedback loop, formed by Brat acting on Mad availability and Nos acting on Brat availability. dMyc is modeled here, but without feedback on other components. The complete system comprises six state variables and 22 parameters (kinetic rates, regulatory constants, and the bound receptor level). Equations were solved numerically using MATLAB® and the Matcont numerical bifurcation analysis software (Dhooge et al., 2003).

The local model equations were expanded further to develop a 3D spatiotemporal model of the germarium. The nondimensionalized equations, including boundary conditions, are given in Supplemental Experimental Procedures. This system considers BMP diffusing from a source boundary at the anterior end of the germarium and binding to receptors at the surface of cells, where signaling and receptor endocytosis occurs. In addition to expanding the previous model spatially, this system considers the spatial distribution of Pum and RBP9, an inhibitor of Bam (Kim-Ha et al., 1999) . We developed partial differential equation balances on extracellular, membrane-bound, and intracellular components. All species are free to diffuse in each subdomain and on the cell membrane, and ligand uptake occurs by the binding of extracellular Dpp to transmembrane receptors, which are internalized as a Dpp-receptor complex. Effects of alternate mechanisms of dMyc activity are considered in the 3D model. In one model the effect of dMyc modulating the rate of BMP-bound receptor endocytosis is tested. For simplicity we assumed a linear relationship between scaled dMyc, added to a constant basal endocytosis rate. To describe the alternate dMyc model, wherein protein synthesis rates for many intracellular processes are upregulated by dMyc, production terms for Mad, Bam, Nos, and Brat linearly depend on the concentration of dMyc. Partial differential equations were solved using COMSOL Multiphysics®. All parameter values were obtained in the literature where available or estimated from similar processes in other systems (see Supplemental Experimental Procedures for details).

## Supplemental Information

Document S1. Five Figures and Supplemental Experimental Procedures
