# Supplementary material for: Brat Promotes Stem Cell Differentiation via Control of a Bistable Switch that Restricts BMP Signaling
Source: Dev Cell. 2011 Jan 18;20(1):72–83. doi: 10.1016/j.devcel.2010.11.019 (PMC3178012; doi:10.1016/j.devcel.2010.11.019)
Supplement: Document S1. Five Figures and Supplemental Experimental Procedures [file mmc1.pdf]

Developmental Cell, Volume *20*

Supplemental Information

Brat Promotes Stem Cell Differentiation via Control of a Bistable Switch that Restricts BMP Signaling

Robin E. Harris, Michael Pargett, Catherine Sutcliffe, David Umulis, and Hilary L. Ashe

Supplemental Information Inventory

Figure S1: Expression of Brat in the *bam* mutant ovary, RNAi and GFP controls for Brat regulation in tissue culture, and the repression of Brat by ectopic Nos expression in ovaries, supporting data in Figure 1.

Figure S2: Additional *brat* mutant alleles have a similar ectopic GSC phenotype to that of the *brat*^{11} allele analysed in Figure 2.

Figure S3: Large scale morphological changes of ovaries expressing different *brat* transgenes analysed in Figures 3 and 4.

Figure S4: Extension of the investigation into Mad repression in Figure 5, showing effective Nos knockdown by RNAi, and that Mad regulation is not through its coding sequence, but is affected by increased presence of Nos.

Figure S5: Information relating to computational modelling shown in Figure 6.

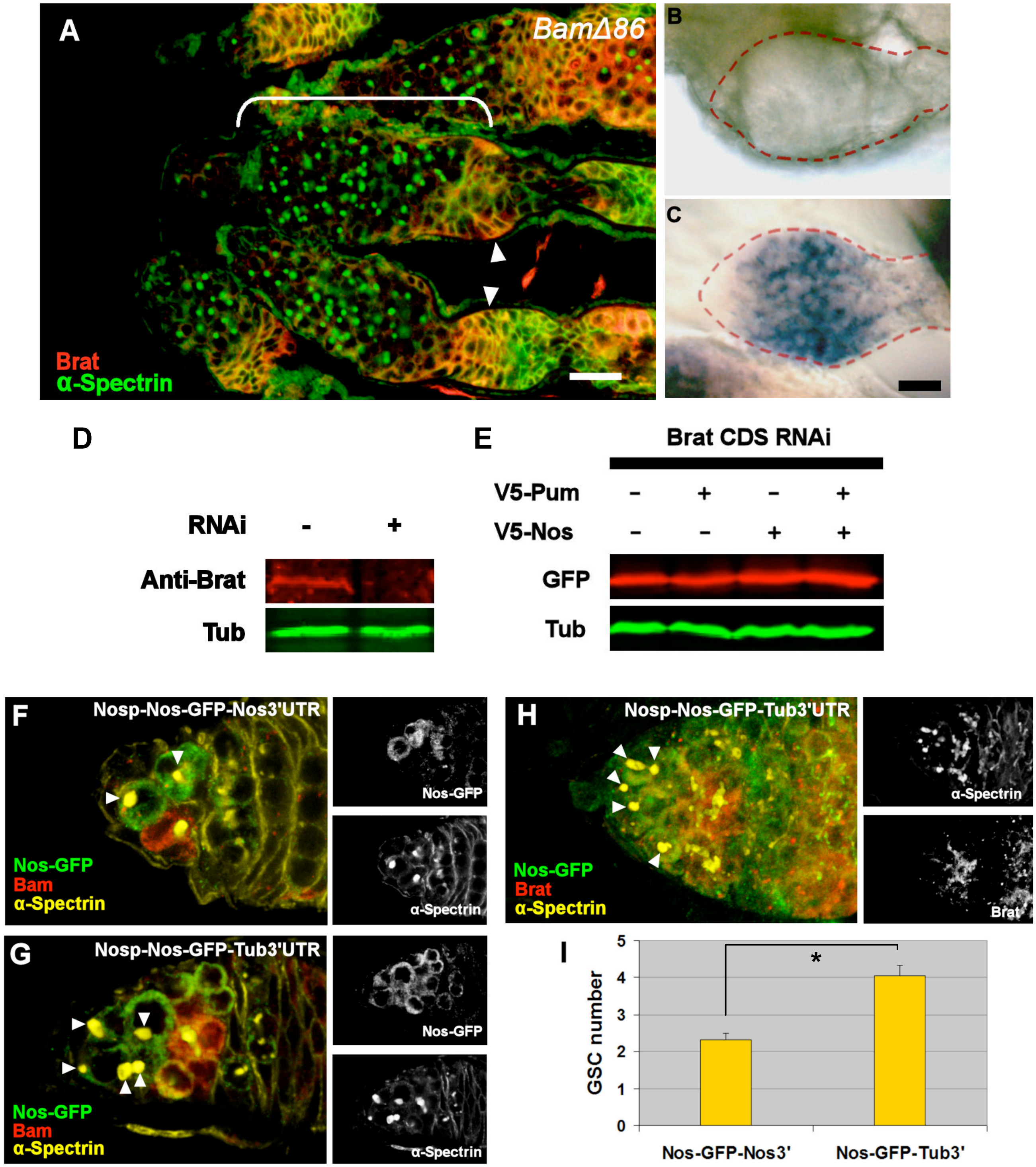

Figure S1, related to Figure 1: Brat expression is downstream of Bam, and is repressed by RNAi in tissue culture and by ectopic Nos in vivo. (A) *bam* mutant germaria contain only undifferentiated GSCs, shown by multiple round spectrosome organelles throughout the germarium, rather than branched fusomes (α-Spectrin, green). Staining with Brat specific

antibodies reveals that Brat protein is absent from these cells, but still expressed in somatic follicle cells (arrowheads), suggesting Brat is expressed as a consequence of Bam. Bracket denotes extent of germarium. Scale bars represent 10μm. (B-C) *In situ* hybridisation of *bam* homozygous ovaries with *brat* sense (B) and antisense (C) probes, showing that *brat* is still transcribed in the ectopic GSCs of the *bam* mutant. Red dashed outline indicates boundary of the germarium. Scale bar represents 20μm. (D) Western blot showing loss of endogenous Brat protein expression following treatment with RNAi targeting *brat*. (E) Western blot analysis reveals there is no change in GFP expression when co-transfected with the regulators Pum and Nos. (F) Expression of a *nos-GFP* transgene bearing the *nos* 3'UTR is regulated normally by Bam in CBs, generating a WT number of GSCs (arrowheads, as in other panels). (G) Expression of a *nos-GFP* transgene bearing the *tubulin* 3'UTR is not downregulated by Bam and is therefore expressed throughout the anterior germarium. The additional Nos and existing Pum repress the differentiation program, including Brat expression (H), within these cells, yielding ectopic GSCs. Decline of Pum levels in more posterior cells (Figure 4C) eventually leads to a loss of Pum-Nos mediated repression, thus allowing differentiation in these cells. (I) Quantitation of GSC number in genotypes in (F) and (G), n=20, error bars are +SEM, $^*p<0.01$.

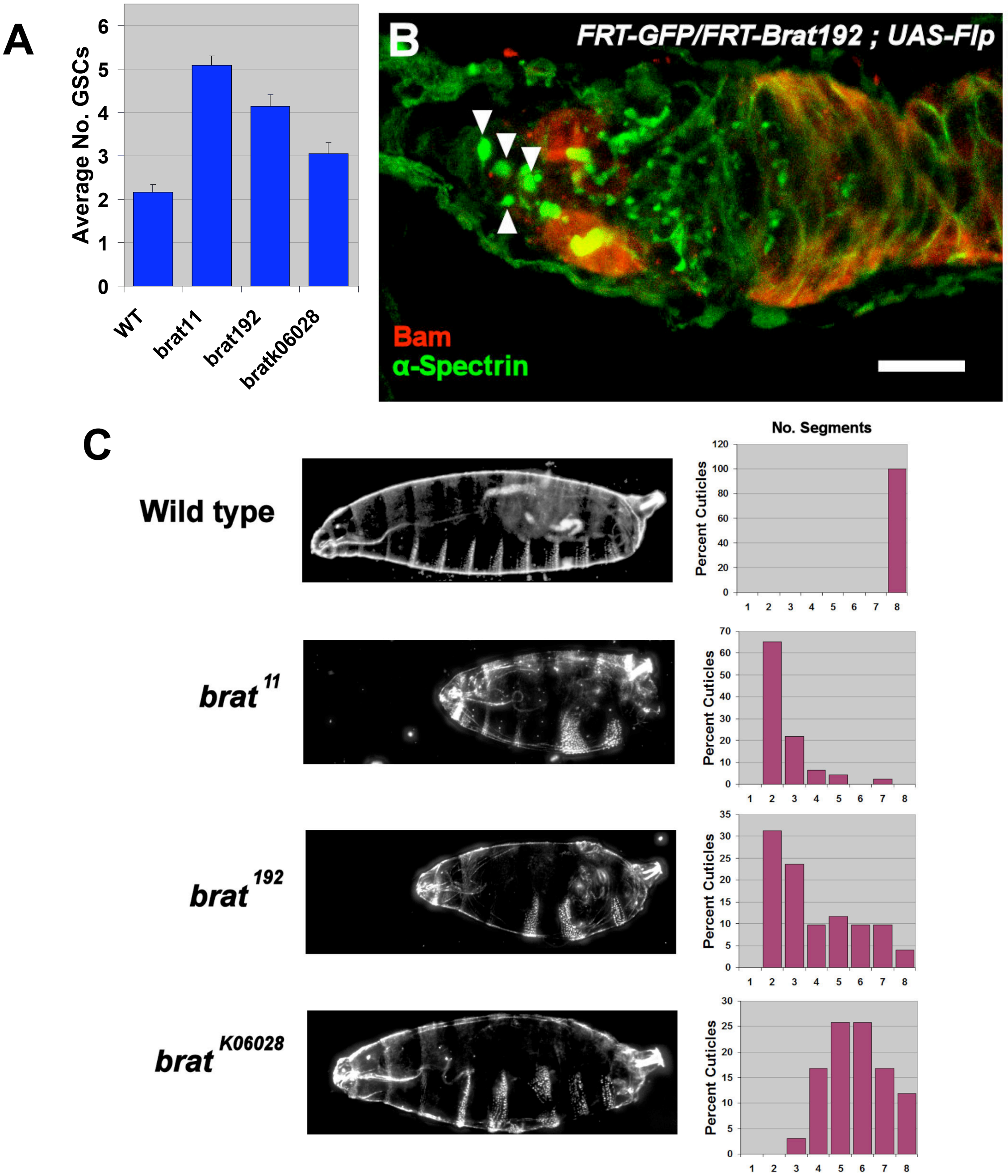

Figure S2, related to Figure 2: Additional Brat alleles have ectopic GSCs. (A) Quantitation of GSC number in *brat* mutants. Difference in GSC number of each mutant allele compared to wild type is statistically significant, $p<0.05$. (B) Representative germarium from ovaries with germline mutant for the strong *brat*^{192} allele. Unlike the mutant phenotype of the Brat family member *mei-P26* (Neumuller et al., 2008), there is no evidence of a "cystocytoma" type expansion of cystocytes at the posterior germarium in any *brat* mutant allele examined. GSCs (arrowheads) are recognized by round fusomes shown by α-Spectrin staining (green) and a lack of Bam (red). Scale bars represent 10μm. (C) *brat* mutants exhibit anterior-posterior patterning defects, and therefore the allelic strength of each *brat* mutant can be evaluated by the number

of segments present in *brat* germline clone embryos. Quantitation of segment number from cuticle preparations (n=50) and a representative embryo cuticle of each genotype is shown. *brat*^{11} and *brat*^{192} demonstrate the strongest defects, most commonly bearing only 2 segments. *brat*^{192} has a greater proportion of weaker defects potentially due to increased paternal rescue. *brat*^{k06028} produces a much milder phenotype. The strength of the alleles is consistent with the nature of each mutation, as *brat*^{11} and *brat*^{192} encode truncations of the Brat protein before the vital C-terminal NHL domain (Arama et al., 2000; Betschinger et al., 2006), while *brat*^{k06028} is an insertion in the 5'UTR that potentially produces unstable mRNA, but still expresses low levels of wild type protein (Arama et al., 2000).

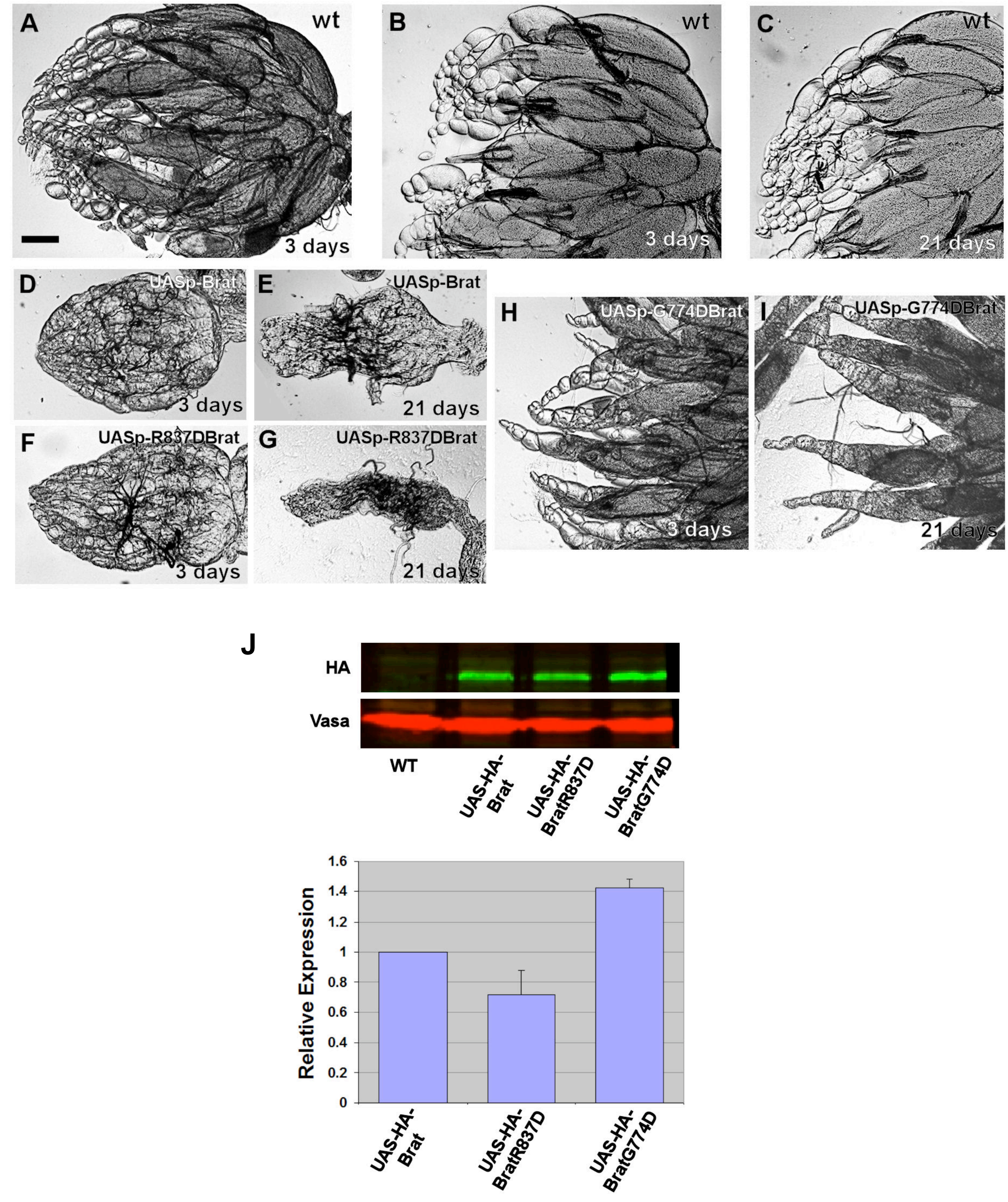

Figure S3, related to Figures 3 and 4: Morphological changes in ovaries expressing different Brat transgenes. All images shown at same magnification, scale bar represents 100µm. (A) Whole mount wild type ovaries grown at 21°C dissected from 3 day old flies, and following mechanical separation to show ovariole structure detail at 3 days (B) and at 21 days

(C). (D) Whole ovary from flies expressing a *UASp-brat* transgene throughout the germline at 3 days. Ovaries initially lack developed embryos, and following 21 days (E) have a shrunken appearance, being devoid of germline cells. Ovaries from flies expressing the *brat*^{R837D} transgene have a similar appearance at both 3 days (F) and 21 days (G). In contrast, ovaries from flies expressing the *brat*^{G774D} transgene have an almost wild type morphology, clearly bearing ovarioles and mature embryos at 3 days (H) which persist until 21 days (I). (J) Brat mutant transgenes are expressed at equivalent levels. Western blot analysis (top) and quantitation (bottom) showing levels of each transgene are expressed at comparable levels in dissected ovaries, measured relative to Vasa expression. n=3, error bars are +SD.

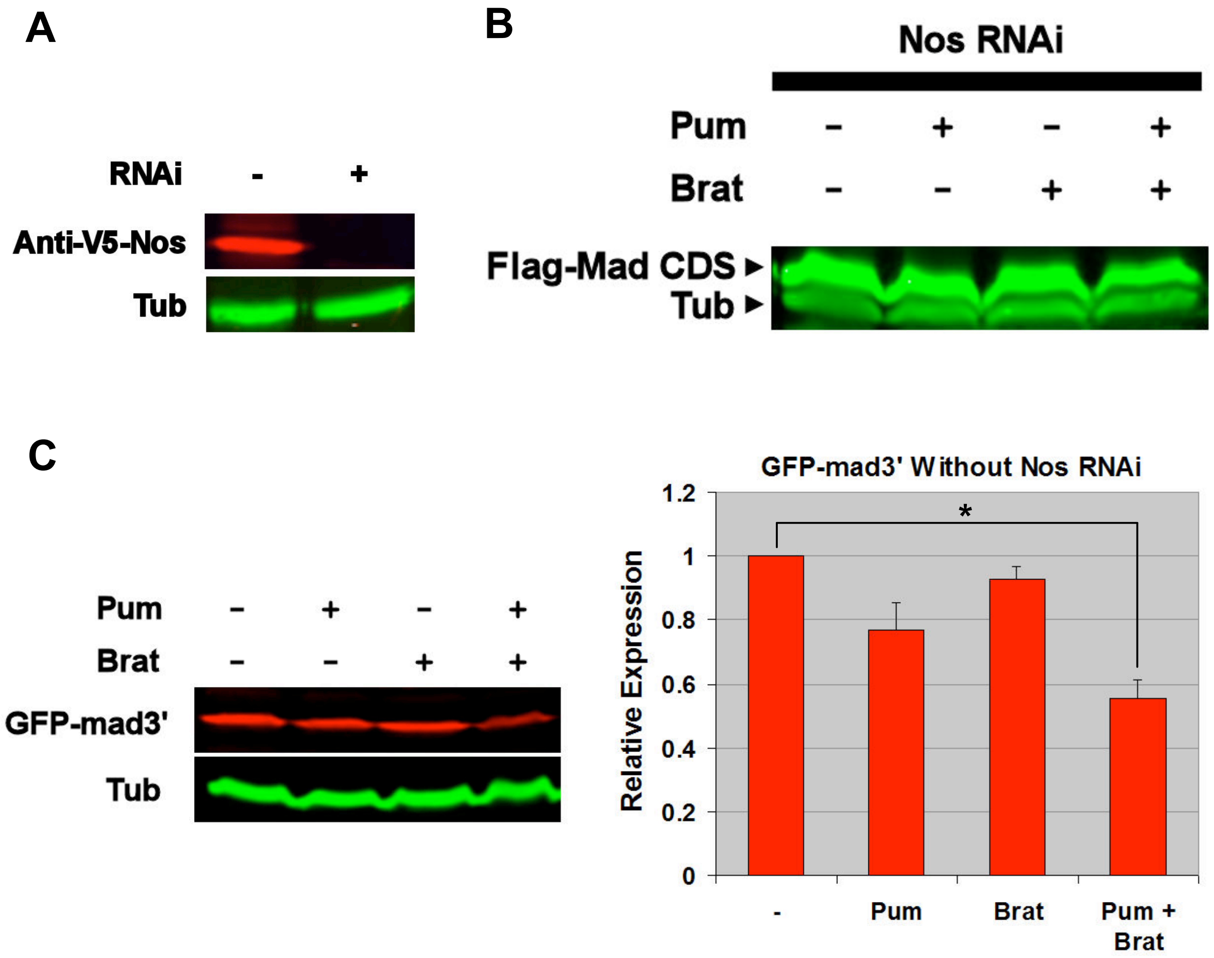

Figure S4, related to Figure 5: Repression of *Mad* is not through its coding sequence, but is affected by Nos levels. (A) Western blot showing loss of transfected Nos following treatment with RNAi. (B) An epitope tagged *mad* coding sequence without UTRs is not regulated by Pum or Brat, consistent with regulation occurring via the *mad* 3'UTR. (C) Removal of *nos* RNAi reduces GFP-mad3' repression. Western blot (left) and quantification (right) showing that an increase in Nos protein through removal of RNAi does not affect the pattern of GFP-mad3' reporter repression by Pum and Brat, but the level of repression is reduced. This is likely due to the Nos protein sequestering Pum, inhibiting the formation of a Pum-Brat repressive complex. In addition, this increased level of Pum-Nos complex can act to repress Brat, thus further inhibiting the GFP-mad3' reported regulation. Quantification shows mean relative expression of independent repeats (n=3), error bars are +SEM, *$p<0.05$.

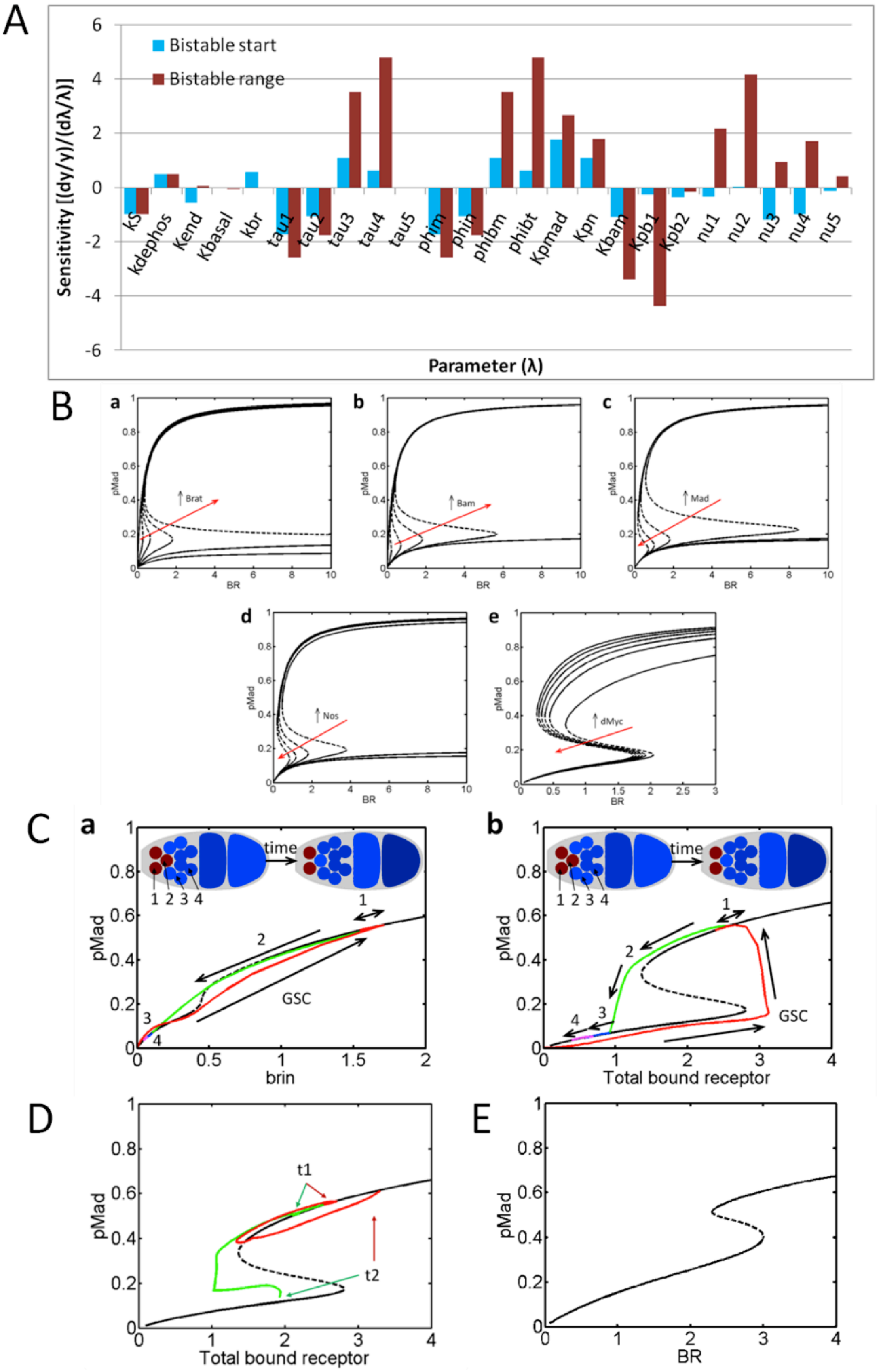
A
Bistable start
Bistable range
Sensitivity [(dγ/γ)/(dλ/λ)]
Parameter (λ)
6
4
2
0
-2
-4
-6
kS
kdephos
Kend
Kbasal
kbr
tau1
tau2
tau3
tau4
tau5
phim
phin
phibm
phibt
Kpmad
Kpn
Kbam
Kpb1
Kpb2
nu1
nu2
nu3
nu4
nu5
B
a
b
c
d
e
pMad
BR
Brat
Bam
Mad
Nos
dMyc
C
a
b
time
1 2 3 4
GSC
pMad
brin
Total bound receptor
D
t1
t2
pMad
Total bound receptor
E
pMad
BR

Figure S5, related to Figure 6: Computational modeling supplemental information. (A) A local sensitivity analysis was performed to investigate the effects of parameter perturbation on the position and range of the bistable region in the BR dimension. Bistable start is defined as the lowest value of BR with multiple equilibria and bistable range the difference between the highest and lowest bistable BR values. Perturbations about the nominal parameter set revealed that system bistability is most sensitive to the four lumped parameters that define the regulatory terms. The lumped parameters are of the form $[pm]\frac{\phi_{pMad}\tau_1}{K_{pMad}}$, yielding the observed correlations among production (φ), degradation (τ), and regulatory (K) parameters. As expected, a qualitative correlation is observed between the lumped regulatory parameters and cooperativity coefficients (ν). Bistability of the system was relatively insensitive to the remaining parameters. (B) To visualize the effects of parameter variation on the system bistability in the model with dMyc regulated endocytosis, families of bifurcation diagrams were plotted with one parameter varied. (a-e) Families of bifurcation curves plotted for Brat, Bam, Mad, Nos and dMyc for various production rates (0, 0.0001, 0.0002, 0.00025, 0.0003 nM*sec-1). All curves are plotted against the bound receptor (BR) parameter. (C) Further views of the progression of cells over time are plotted against the internalized bound receptor concentration (brin) and against the total bound receptor. The cell trajectories for a GSC, CB, 2-cell and 4-cell cyst are shown plotted against internalized bound receptor (a) and total bound receptor, the sum of both surface and internalized bound receptor (b). The total bound receptor quantity gives the most direct approximation of BMP signaling to the cell by representing both surface-bound and internalized signal sources. (D) As a second visualization of the progress of a 4xdMyc clone over time, the cell trajectories for the clone and the adjacent wild type GSC were plotted through time point t2 (since the positional definitions change afterward, with the displacement of the wild type cell from the niche). The two GSCs from Figure 6f follow different paths, resulting in differentiation of the wild type cell. The 4xdMyc clone (red trajectory) varies only slightly by t1, where the wild type cell's (green trajectory) pMad level decreases significantly. The trajectories diverge further with the decrease and subsequent return of Dpp production until, at t2, the clone is on the high-pMad branch while the wild type cell has fallen to the low-pMad branch. (E) Bifurcation diagram for the alternate dMyc model. The alternate model still supports bistability, though for a different region of the parameter space, illustrated by the bifurcation diagram.

Supplementary Experimental Procedures

Fly stocks

The following alleles were also used in this study: *BamΔ89* (Bloomington Stock Center) *nos*P-Nos-GFP-*nos* 3′-UTR and *nos*P-Nos-GFP-*tub* 3′-UTR (Li et al., 2009), $brat^{192}$ (Luschnig et al., 2004) and $brat^{K06028}$ (Kyoto Stock Centre). Mutant germline tissue in adult ovaries and germline clone embryos were generated as described in experimental procedures using *brat* alleles.

Immunofluorescence

Adults were dissected after maturing on yeast and apple juice agar plates. Ovaries were fixed and stained using standard techniques (detailed methods available on request). The following primary antibodies were used: mouse anti-α-Spectrin (1:20, DSHB), rabbit anti-Vasa (Hay et al., 1990) (1:500), rabbit anti-Brat (Betschinger et al., 2006) (1:250), rat anti-Bam (McKearin and Ohlstein, 1995) (1:1000), rabbit anti-β-Galactosidase (1:1000, Cappel), rabbit anti-GFP (1:500, Abcam), mouse anti-HA (1:500, Roche), mouse anti-Myc (1:500, Roche), mouse anti-dMyc (1:500). The following secondary antibodies were used: goat anti-mouse and anti-rabbit FITC (1:100, Jackson Labs), goat anti-mouse Alexa-555, anti-mouse and anti-rabbit Alexa 594 (1:500 Invitrogen). Samples were counterstained with Dapi (1:300, Invitrogen), mounted using Prolong Anti-Fade Mounting Kit (Invitrogen) and visualised with a Leica SP5 Confocal Microscope, either as single focal plane or compiled Z-stacks. Cell death was detected via TUNEL assay, using *in situ* Cell Death Detection Kit, TMR Red (Roche) according to manufacturers' instructions, viability staining was performed using Image-iT DEAD green (Invitrogen) according to manufacturers' instructions. As a positive control for cell death *hs-grim* adult flies were heat shocked for 30 minutes at 37°C, dissected after an hour and stained immediately. RNA *in situ* was performed using standard methods, using E.L.F. substrate (Invitrogen) according to manufacturers' instructions.

RNAi

dsRNA was generated by MegaScript T7 Kit (Ambion), using fragments of *brat* (BDGP: LD16270) and *nos* amplified from genomic DNA as templates, designed using the E-RNAi tool (http://www.dkfz.de/signaling/e-rnai3/) to prevent off target effects. 10ug of dsRNA was added to 2mls serum free cells per sample, followed by addition of serum after 30 mins.

Transgenic expression assessment

Level of *brat* transgene expression was measured in adult ovaries by dissecting 20 ovaries from newly eclosed flies of each genotype, removing embryos that were present and homogenizing the remaining germarium tissue in 100μl sample buffer. 30μl of each preparation were analysed by western blot, and to account for any loss of germline due to differentiation caused by ectopic *brat* expression, levels were equalized to the germline marker Vasa.

Cuticle preparations

Flies were allowed to lay for 3 hours and embryos aged for 18 hours at 25°C, after which they were collected by washing in 0.1% Triton-X, bleached for 2 minutes, rinsed thoroughly in distilled water, and added to a 1:1 heptane/MeOH solution. Samples were shaken vigorously for 30 seconds, allowed to settle and the supernatant and floating embryos removed. Samples were rinsed 3 times in MeOH, 2 times in 1:1 MeOH/0.1% Triton-X and pipetted gently onto a slide. Excess liquid was removed with blotting paper, several drops of 3:1 Lactic acid/H_2O added and a coverslip gently applied. Cuticles were cleared overnight at 65°C, slides sealed with nail varnish and images taken with a Leica compound microscope.

Mathematical Modelling

To investigate local and spatial interactions mediated by Brat repression, we developed: (1) a local model of intracellular interactions, analyzed in steady state and (2) a three dimensional finite element model of the germarium, including representations of stem cells and cystoblasts, as well representations of two-four, four-eight, sixteen cell cysts in region 2A and encapsulated cysts in the posterior germarium.

Equations for the local model are given below (1-6). The local analysis is performed by continuation of the system across values of a bound receptor, which provides the input level for signaling, the concentration of which is represented as $[BR]$ below. In the local system where BR is treated as a parameter, it is capitalized. When it is a variable solved for in the spatial model it is given as lowercase *br*.

$$\frac{d\left[pMad\right]}{dt} = k_S\left[BR\right]\left[Mad\right] - k_{dephos}\left[pMad\right] - k_{deg1}\left[pMad\right] \quad (1)$$

$$\frac{d\left[Mad\right]}{dt} = \frac{\phi_{Mad}}{1+\left[\frac{\left[Brat\right]}{K_{PB1}}\right]^{v1}} - k_S\left[BR\right]\left[Mad\right] + k_{dephos}\left[pMad\right] - k_{deg1}\left[Mad\right] \quad (2)$$

$$\frac{d\left[Nos\right]}{dt} = \frac{\phi_{Nos}}{1+\left[\frac{\left[Bam\right]}{K_{Bam}}\right]^{v2}} - k_{deg2}\left[Nos\right] \quad (3)$$

$$\frac{d\left[Bam\right]}{dt} = \frac{\phi_{Bam}}{1+\left[\frac{\left[pMad\right]}{K_{pMad}}\right]^{v3}} - k_{deg3}\left[Bam\right] \quad (4)$$

$$\frac{d\left[Brat\right]}{dt} = \frac{\phi_{Brat}}{1+\left[\frac{\left[Nos\right]}{K_{PN}}\right]^{v4}} - k_{deg4}\left[Brat\right] \quad (5)$$

$$\frac{d\left[dMyc\right]}{dt} = \frac{\phi_{dMyc}}{1+\left[\frac{\left[Brat\right]}{K_{PB2}}\right]^{v5}} - k_{deg5}\left[dMyc\right] \quad (6)$$

$\left[Mad\right]$, $\left[pMad\right]$, $\left[Nos\right]$, $\left[Bam\right]$, $\left[Brat\right]$, and $\left[dMyc\right]$ are the concentrations of the corresponding protein components in a cell. Parameters are defined in Table 1. The k_{deg} parameters are degradation rate constants for each of the proteins.

The equations were further simplified by non-dimensionalizing the concentration of Mad, Nos, Bam, and Brat by the maximum level the equations allow for each component. After this reduction, the maximum level of *mad, nos, bam,* and *brat* is 1.

$$\left[m\right]=\left[Mad\right]\frac{k_{deg1}}{\phi_{Mad}};\ \left[pm\right]=\left[pMad\right]\frac{k_{deg1}}{\phi_{Mad}};\ \left[n\right]=\left[Nos\right]\frac{k_{deg2}}{\phi_{Nos}};$$
$$\left[bm\right]=\left[Bam\right]\frac{k_{deg3}}{\phi_{Bam}};\ \left[bt\right]=\left[Brat\right]\frac{k_{deg4}}{\phi_{Brat}};\ \left[dmyc\right]=\left[dMyc\right]\frac{k_{deg5}}{\phi_{dMyc}} \quad (7)$$

$$\tau_1 = k_{deg1}{}^{-1};\ \tau_2 = k_{deg2}{}^{-1};\ \tau_3 = k_{deg3}{}^{-1};\ \tau_4 = k_{deg4}{}^{-1};\ \tau_5 = k_{deg5}{}^{-1} \quad (8)$$

Furthermore, the equations for pMad [1] and Mad [2] were added to create a new equation for the total amount of Mad ($\left[m_T\right]$) in the system, yielding the following equations.

$$\frac{d\left[pm\right]}{dt} = k_S\left[BR\right]\left[m\right] - k_{dephos}\left[pm\right] - \tau_1^{-1}\left[pm\right] \qquad \left[pm\right]\in\left[0,1\right] \quad (9)$$

$$\frac{d\left[m_T\right]}{dt} = \tau_1^{-1}\left[\frac{1}{1+\left[\left[bt\right]\frac{\phi_{Brat}\tau_4}{K_{PB1}}\right]^{\upsilon 1}} - \left[m_T\right]\right] \qquad \left[m_T\right]\in\left[0,1\right] \quad (10)$$

$$\left[m_T\right] = \left[m\right] + \left[pm\right] \quad (11)$$

$$\frac{d\left[n\right]}{dt} = \tau_2^{-1}\left[\frac{1}{1+\left[\left[bm\right]\frac{\phi_{Bam}\tau_3}{K_{Bam}}\right]^{\upsilon 2}} - \left[n\right]\right] \qquad \left[n\right]\in\left[0,1\right] \quad (12)$$

$$\frac{d\left[bm\right]}{dt} = \tau_3^{-1}\left[\frac{1}{1+\left[\left[pm\right]\frac{\phi_{pMad}\tau_1}{K_{pMad}}\right]^{\upsilon 3}} - \left[bm\right]\right] \qquad \left[bm\right]\in\left[0,1\right] \quad (13)$$

$$\frac{d\left[bt\right]}{dt} = \tau_4^{-1}\left[\frac{1}{1+\left[\left[n\right]\frac{\phi_{Nos}\tau_2}{K_{PN}}\right]^{\upsilon 4}} - \left[bt\right]\right] \qquad \left[bt\right]\in\left[0,1\right] \quad (14)$$

$$\frac{d[dmyc]}{dt} = \tau_5^{-1}\left[\frac{1}{1+\left[[bt]\frac{\phi_{Brat}\tau_4}{K_{PB2}}\right]^{\upsilon 5}} - [dmyc]\right] \qquad [dmyc]\in[0,1] \tag{15}$$

With this network we investigated the steady-state behaviour of the level of each of the components with respect to changes in the level of the extracellular BMP-bound receptor (BR). The steady-state behaviour depends on only 7 parameter groups and the 5 Hill coefficient cooperativity parameters. Hill coefficients were set to 2 to represent weak cooperativity in repressor binding for both transcription factors and for mRNA repression. Analysis was performed by continuation of equilibrium points, across values of the parameter $[BR]$, using the MatCont 2.5.1 package with Matlab (R2009a).

Table S1: Model Parameters

Parameter	Symbol	Value
Bound Receptor	$[BR]$	*variable*
Signaling Constant	k_S	2e-4 $nM^{-1}*sec^{-1}$
Dephosphorylation Rate Parameter	k_{dephos}	2e-4 sec^{-1}
Protein Degradation Parameters	k_1, k_2, k_3, k_4, k_5	2e-4 sec^{-1}
Repression Hill Coefficients	$\upsilon 1$, $\upsilon 2$, $\upsilon 3$, $\upsilon 4$, $\upsilon 5$	2
Pum-Brat Half-Occupation Concentration (re: *mad*)	K_{PB1}	0.5nM
Pum-Brat Half-Occupation Concentration (re: *dmyc*)	K_{PB2}	0.2nM
Bam Half-Occupation Concentration	K_{Bam}	0.2nM
pMad Half-Occupation Concentration	K_{pMad}	0.2nM
Pum-Nos Half-Occupation Concentration	K_{PN}	0.2nM
Maximum Production Rates	ϕ_{Brat}, ϕ_{Bam}, ϕ_{Nos}, ϕ_{pMad}	2e-4 $nM*sec^{-1}$

The local model equations were expanded to develop a three dimensional spatiotemporal model of the germarium, for which the non-dimensionalized equations are given

below. The BMP source is defined as the anterior boundary. This model includes dMyc-mediated endocytosis, such that the rate of endocytosis is k_e=*f(dMyc)* with units sec^{-1}. Note that in the scaled equations, the repression behaviour depends on the balance between production, decay, and the saturation parameter K_i. While we have rough estimates for the decay rates, we do not have an estimate for production or the saturation parameter. However, the dimensionless quantity: (production/decay * $1/K_i)^{-1}$ is the scaled half-maximal saturation parameter, which is between 0.2 and 0.5 for the species in the model.

$$\begin{aligned}
&\frac{\partial[bmp]}{\partial t}=\frac{D_{bmp}}{L^2}\nabla^2 bmp-k_{ext}[bmp] \\
&\boldsymbol{n}\bullet\left(\frac{D_{bmp}}{L}\nabla[K\cdot bmp]\right)=-k_{on}[bmp][r]+k_{off}[br]\ \ on\ cell\ surface \\
&\boldsymbol{n}\bullet\left(\frac{D_{bmp}}{L}\nabla[bmp]\right)=\phi_{bmp}\ \ at\ source \\
&\boldsymbol{n}\bullet\left(\nabla[bmp]\right)=0\ elsewhere
\end{aligned} \tag{16}$$

$$\frac{\partial[br]}{\partial t}=\frac{D_{br}}{L^2}\nabla^2[br]+k_{on}[bmp][r]-k_{off}[br]-k_{end}\phi_{dMyc}\tau_5[dmyc][br]-k_{basal}[br] \tag{17}$$

$$r=R_{tot}-br \tag{18}$$

$$\begin{aligned}
&\frac{d[brin]}{dt}=\frac{D_{brin}}{L^2}\nabla^2[brin]-k_{br}[brin] \\
&\boldsymbol{n}\bullet\left(\frac{D_{brin}}{L}\nabla[K\cdot brin]\right)=k_{end}\phi_{dMyc}\tau_5[dmyc][br]+k_{basal}[br]
\end{aligned} \tag{19}$$

$$\begin{aligned}
&\frac{d[pm]}{dt}=\frac{D_{pm}}{L^2}\nabla^2[pm]+k_S[brin][m]-k_{dephos}[pm]-\tau_1^{-1}[pm] \\
&\boldsymbol{n}\bullet\left(\frac{D_{pm}}{L}\nabla[K\cdot pm]\right)=k_S[br][m]
\end{aligned} \tag{20}$$

$$\frac{d[m_T]}{dt}=\frac{D_m}{L^2}\nabla^2 m_T+\tau_1^{-1}\left[\frac{1}{1+\left[[bt]\frac{\phi_{Brat}\tau_4}{K_{PB1}}\right]^{\upsilon 1}}-[m_T]\right] \tag{21}$$

$$\boldsymbol{n}\bullet\left(\frac{D_m}{L}\nabla\left[m_T\right]\right)=0$$

$$\left[m_T\right]=\left[m\right]+\left[pm\right] \tag{22}$$

$$\frac{d\left[n\right]}{dt}=\frac{D_n}{L^2}\nabla^2 n+\tau_2^{-1}\left[\frac{1}{1+\left[\left[bm\right]\frac{\phi_{Bam}\tau_3}{K_{Bam}}\right]^{\upsilon 2}}-\left[n\right]\right] \tag{23}$$

$$\boldsymbol{n}\bullet\left(\frac{D_n}{L}\nabla\left[n\right]\right)=0$$

$$\frac{d\left[bm\right]}{dt}=\frac{D_{bm}}{L^2}\nabla^2 bm+\tau_3^{-1}\left[\frac{1}{1+\left[\left[pm\right]\frac{\phi_{pMad}\tau_1}{K_{pMad}}\right]^{\upsilon 3}+10\left[rpb9\right]}-\left[bm\right]\right] \tag{24}$$

$$\boldsymbol{n}\bullet\left(\frac{D_{bm}}{L}\nabla\left[bm\right]\right)=0$$

$$\frac{d\left[bt\right]}{dt}=\frac{D_{bt}}{L^2}\nabla^2 bt+\tau_4^{-1}\left[\frac{1}{1+\left[\left[n\right]\frac{\phi_{Nos}\left[pum\right]\tau_2}{K_{PN}}\right]^{\upsilon 4}}-\left[bt\right]\right] \tag{25}$$

$$\boldsymbol{n}\bullet\left(\frac{D_{bt}}{L}\nabla\left[bt\right]\right)=0$$

$$\frac{d\left[dmyc\right]}{dt}=\frac{D_{dmyc}}{L^2}\nabla^2 dmyc+\tau_5^{-1}\left[\frac{1}{1+\left[\left[bt\right]\frac{\phi_{Brat}\tau_4}{K_{PB2}}\right]^{\upsilon 5}}-\left[dmyc\right]\right] \tag{26}$$

$$\boldsymbol{n}\bullet\left(\frac{D_{dmyc}}{L}\nabla\left[dmyc\right]\right)=0$$

Defining boundary conditions for all applicable equations, $\boldsymbol{n}$ is the outward facing normal taken relative to the domain for which the equations are written. For $[br]$, a weak form solution is applied along the cell boundaries. Both Pum and RBP9 are implemented as functions of position x only (the distance from the anterior end, along the anterior-posterior axis). We assumed that the level of Pumilio is maximal near the anterior of the germarium near the niche and decays toward the posterior and that RBP9 is active in the region 2A (after x>0.5) and off elsewhere. Pum and RBP9 are specified to approximate expression as observed in experimentation (Kim-Ha et al., 1999). Hs is a smoothed Heaviside step function (Comsol's flsmhs). A unit conversion parameter, defined as $K \equiv 10^{-15}\frac{liter}{\mu m^3}\cdot 10^{-9}\frac{mol}{nmol}\cdot 6.022\cdot 10^{23}\frac{molecules}{mol}$, is used to establish boundary conditions with flux to or from a surface. Parameters of this system are defined in Table S2, below.

Table S2: Three Dimensional Model Parameters

Parameter	Symbol	Value
Model Length of Germarium	L	50 microns
BMP Influx Rate	ϕ_{bmp}	7.7e-5 micron*nM**sec*$^{-1}$
BMP Diffusion Coefficient	D_{bmp}	0.3 microns^2**sec*$^{-1}$
Diffusion Coefficient	D_{brin}, D_{pm}, D_m, D_n, D_{bm}, D_{bt}, D_{dmyc}	10 microns^2**sec*$^{-1}$
BMP Diffusion Coefficient	D_{br}	0.1 microns^2**sec*$^{-1}$
Unit Conversion Parameter	K	$\approx$0.6 molecules*nM^{-1}*microns^{-3}
BMP Degradation Rate Parameter	k_{ext}	0
BMP-Receptor Association Rate Parameter	k_{on}	2e-4 nM^{-1}**sec*$^{-1}$
BMP-Receptor Dissociation Rate Parameter	k_{off}	6e-4 *sec*$^{-1}$
Total Number of Receptors	R_{tot}	100 molecules*micron^{-2}
Signaling Parameter	k_S	2e-4 nM^{-1}**sec*$^{-1}$
Dephosphorylation Rate Parameter	k_{dephos}	2e-4 *sec*$^{-1}$
Protein Degradation Parameters	k_1, k_2, k_3, k_4, k_5	2e-4 *sec*$^{-1}$
Repression Hill Coefficients	$\upsilon 1$, $\upsilon 2$, $\upsilon 3$, $\upsilon 4$, $\upsilon 5$	2
Pum-Brat Half-Occupation Concentration (re: *mad*)	K_{PB1}	0.5nM

Pum-Brat Half-Occupation Concentration (re: *dmyc*)	K_{PB2}	0.2nM
Bam Half-Occupation Concentration	K_{Bam}	0.2nM
pMad Half-Occupation Concentration	K_{pMad}	0.2nM
Pum-Nos Half-Occupation Concentration	K_{PN}	0.2nM
Maximum Production Rates	ϕ_{Brat}, ϕ_{Bam}, ϕ_{Nos}, ϕ_{pMad}	2e-4 nM*sec^{-1}
Endocytosis Rate Parameter	k_{end}	4e-4 $nM^{-1}sec^{-1}$
Basal Endocytosis Rate Parameter	k_{basal}	8e-6 sec^{-1}
Endocytosed Receptor Degradation Parameter	k_{br}	2e-4 sec^{-1}

The system is solved via the Galerkin Finite Element method using the broadly available Comsol Multiphysics software package (version 3.5a). The model is implemented on a geometry approximating the *Drosophila* germarium, including cell subdomains within a whole-germarium subdomain (Figure SEP1). BMP is modeled within the germarium subdomain, outside of the cell subdomains; r and br are modeled on the boundaries of the cell subdomains; all other components are modeled within each cell subdomain.

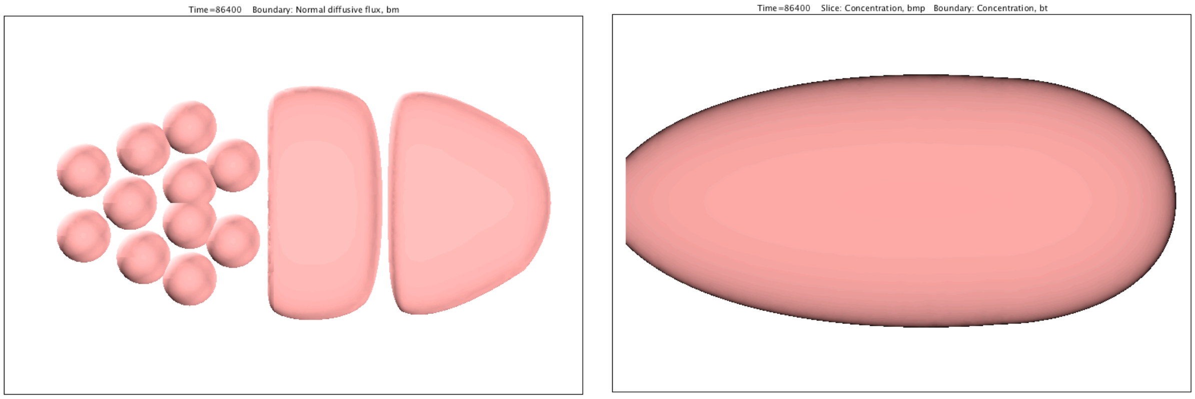

Figure SEP1. Subdomains of the three dimensional model geometry. (Left) Cell model volumes, representing GSCs, a pre-cystoblast/cystoblast, 2-cell cysts, a 4-cell cyst, an 8-cell cysts and a sixteen cell cyst, from left to right. (Right) Model volume of the outer germarium structure, which contains the cells.

Specific parameter value determination

The diffusion rate of free, extracellular Dpp was approximated by two measured rates for different molecules involved in embryo development: Dpp in the wing imaginal disc (Kicheva et al., 2007), and Bcd in the syncytial embryo (Gregor et al., 2007) have measured diffusion rates that range from 0.1-0.3 microns^2/second. Intracellular diffusion rates were assumed to be greater than intracellular transport rates, which allows for easier analysis of the model and direct correspondence between the local cellular model analysis and the full 3D germarium model. The diffusion of receptors in the membrane of a cell is approximated as 0.1 microns^2/second, which is in the range of other transmembrane receptors (Lauffenburger and Linderman, 1993).

Kinetic parameters for Dpp binding to type I receptor fall in the middle of the expected range (Umulis et al., 2009) and lead to a dissociation constant of 3nM. Measured dissociation constants typically fall between 0.5 and 10nM for the binding between BMPs and type I BMP receptors.

Kinetic parameters for the intracellular network have not been carefully measured to date. We estimated the phosphorylation rate for surface localized BMP-bound receptors and internalized bound receptors as k_S = 2e-4nM^{-1}*sec^{-1}, which is nearly the same as estimated values for Smad2 phosphorylation in HaCaT cell culture stimulated with TGF-β k_{phos}~3e-4nM^{-1}sec^{-1} (Schmierer et al., 2008). For our initial screen, we selected a receptor density of 100 receptors/square micron or a total of about 7000 receptors per cell, which is in the range of expected values (Lauffenburger and Linderman, 1993) for other systems. All protein degradation parameters were selected to be in the range of typical protein lifetimes and in accordance with the timescale for processes in the germarium. Production rates for intracellular molecules were selected so that the steady-state level of intracellular components is one nM, but the specific values for decay and production do not change the regulation inherent in the network as can be easily seen in the dimensionless forms of the equations. The regulation depends on the ratio of maximum concentration for a particular species to the half maximal concentration hill parameter ($\phi_x \tau_x / K_x$).

Alternate dMyc mechanism

To describe the alternate dMyc model, wherein protein synthesis rates for many intracellular processes are upregulated by dMyc, production terms for Mad, Bam, Nos and Brat were modified by the concentration of dMyc. For example, ϕ_{Brat} was replaced with

$\phi_{Brat}(1+Kpsyn[dmyc])$, where $Kpsyn$ is a parameter describing the strength of upregulation by dMyc, representing the maximum percentage increase in expression. For unit consistency with the endocytosis dMyc mechanism, dimensionless results are shown from [0,1] for pMad in Figure 6. The nondimensionalization introduces a nonlinearity in the mapping of colorscale to absolute scale by 1 + *Kpsyn*[*dmyc*] that largely affects low signaling cells near the posterior of the germarium without affecting the qualitative interpretation of cell state in the dMyc synthesis model. This substitution gives non-dimensionalized equations of the form,

$$\frac{d[m_T]}{dt} = \tau_1^{-1}\left[\frac{1}{1+\left[[bt]\frac{\phi_{Brat}(1+Kpsyn[dmyc])\tau_4}{K_{PB1}}\right]^{v1}} - [m_T]\right] \qquad [m_T]\in[0,1]$$

The illustrative parameter set for the alternate dMyc model was chosen to fit the qualitative observations from experimental data. Parameters that differ from the dMyc-mediated endocytosis model are given in Table S3.

Table S3: Parameters adjusted for the alternate dMyc model

Parameter	Symbol	Value
BMP Influx Rate	ϕ_{bmp}	7e-5 micron*nM*sec^{-1}
Pum-Brat Half-Occupation Concentration (re: *mad*)	K_{PB1}	0.46nM
Bam Half-Occupation Concentration	K_{Bam}	0.58nM
pMad Half-Occupation Concentration	K_{pMad}	0.86nM
Production Rate Regulation	$Kpsyn$	2
Basal Endocytosis Rate Parameter	k_{basal}	2.4e-5 sec^{-1}
Endocytosed Receptor Degradation Parameter	k_{br}	4e-4 sec^{-1}

Supplementary References

Arama, E., Dickman, D., Kimchie, Z., Shearn, A., and Lev, Z. (2000). Mutations in the beta-propeller domain of the Drosophila brain tumor (brat) protein induce neoplasm in the larval brain. Oncogene *19*, 3706-3716.

Betschinger, J., Mechtler, K., and Knoblich, J.A. (2006). Asymmetric segregation of the tumor suppressor brat regulates self-renewal in Drosophila neural stem cells. Cell *124*, 1241-1253.

Gregor, T., Tank, D.W., Wieschaus, E.F., and Bialek, W. (2007). Probing the limits to positional information. Cell *130*, 153-164.

Hay, B., Jan, L.Y., and Jan, Y.N. (1990). Localization of vasa, a component of Drosophila polar granules, in maternal-effect mutants that alter embryonic anteroposterior polarity. Development (Cambridge, England) *109*, 425-433.

Kicheva, A., Pantazis, P., Bollenbach, T., Kalaidzidis, Y., Bittig, T., Julicher, F., and Gonzalez-Gaitan, M. (2007). Kinetics of morphogen gradient formation. Science *315*, 521-525.

Kim-Ha, J., Kim, J., and Kim, Y.J. (1999). Requirement of RBP9, a Drosophila Hu homolog, for regulation of cystocyte differentiation and oocyte determination during oogenesis. Mol Cell Biol *19*, 2505-2514.

Lauffenburger, D., and Linderman, J. (1993). Receptors: Models for Binding, Trafficking, and Signaling (Oxford University Press, New York).

Li, Y., Minor, N.T., Park, J.K., McKearin, D.M., and Maines, J.Z. (2009). Bam and Bgcn antagonize Nanos-dependent germ-line stem cell maintenance. Proc Natl Acad Sci U S A *106*, 9304-9309.

Luschnig, S., Moussian, B., Krauss, J., Desjeux, I., Perkovic, J., and Nusslein-Volhard, C. (2004). An F1 genetic screen for maternal-effect mutations affecting embryonic pattern formation in Drosophila melanogaster. Genetics *167*, 325-342.

McKearin, D., and Ohlstein, B. (1995). A role for the Drosophila bag-of-marbles protein in the differentiation of cystoblasts from germline stem cells. Development (Cambridge, England) *121*, 2937-2947.

Neumuller, R.A., Betschinger, J., Fischer, A., Bushati, N., Poernbacher, I., Mechtler, K., Cohen, S.M., and Knoblich, J.A. (2008). Mei-P26 regulates microRNAs and cell growth in the Drosophila ovarian stem cell lineage. Nature *454*, 241-245.

Schmierer, B., Tournier, A.L., Bates, P.A., and Hill, C.S. (2008). Mathematical modeling identifies Smad nucleocytoplasmic shuttling as a dynamic signal-interpreting system. Proc Natl Acad Sci U S A *105*, 6608-6613.

Umulis, D., O'Connor, M.B., and Blair, S.S. (2009). The extracellular regulation of bone morphogenetic protein signaling. Development *136*, 3715-3728.
